# Rotational Grazing Supports Coexistence of the Endangered Pygmy Bluetongue and Livestock on a Productive Agricultural Property

**DOI:** 10.1002/ece3.72716

**Published:** 2025-12-16

**Authors:** Kimberley H. Michael, Patrick Michael, Ryan Baring, Michael G. Gardner

**Affiliations:** ^1^ College of Science and Engineering Flinders University Adelaide South Australia Australia; ^2^ School of Health Sciences Swinburne University Melbourne Victoria Australia

**Keywords:** disturbance ecology, habitat management, livestock grazing, rangeland, reptile conservation, rotational grazing, sustainable agriculture

## Abstract

Agricultural grasslands have the potential to complement conservation initiatives whilst maintaining productivity. A shift from set‐stock continuous grazing to rotational grazing has the capacity to improve ecosystem function to support both biodiversity and livestock production. Using a ‘before’ and ‘after’ comparative design, we assessed the responses of endangered pygmy bluetongue lizards and vegetation structure to set‐stock, rotational and grazing exclusion on a landscape‐scale. To implement our design, we erected new fence lines to divide an existing set‐stock paddock to become rotationally grazed while the landowner managed stock levels conducive to productivity. We found vegetation cover increased in grazing exclusion and experimental grazing areas and lizards responded positively to this increase. The implementation of rotational grazing was beneficial for lizards. The increase in vegetation cover will likely also be conducive for livestock productivity. Our study demonstrates there are potential mutual benefits, economically and ecologically, for landowners and conservationists to implement rotational grazing.

## Graphical abstract

This research assessed the responses of endangered pygmy bluetongue lizards and vegetation structure to set‐stock, rotational and grazing exclusion on a landscape‐scale. The study demonstrated there are potential mutual benefits, economically and ecologically, for landowners and conservationists to implement rotational grazing.

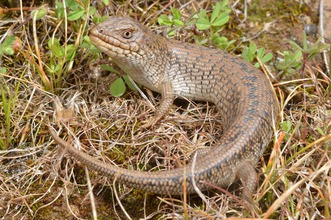

## 1 Introduction

Livestock grazing is globally one of the most widespread land uses and influences biotic processes that affect soil, fauna and flora communities. The basic principles of sustainable grazing management focus on ensuring stocking rates do not exceed the carrying capacity of the land (O'Reagain et al. 2014). Healthy livestock‐grazed grasslands are more productive, stable and resilient and thus provide greater profits and abundant ecosystem services than those in poorer conditions (Teague et al. 2013; Möhrle et al. 2024). The optimal grazing level to maintain livestock productivity and ecosystem richness and function remains unclear, despite extensive manipulative and mensurative research and will vary in different contexts (Eldridge et al. 2016). With livestock grazing expected to intensify and agroecosystems increasingly relied upon as ‘off‐reserve’ conservation areas (Fischer 2011; Neilly et al. 2016), understanding native fauna responses to moderate grazing within productive systems is essential. Managing productive livestock‐grazed grasslands to serve a dual purpose (i.e., food production and conservation) is a key challenge (Neilly et al. 2016).

Declining grassland productivity and shifts in farmer land management perspectives to also encompass native vegetation restoration have influenced long‐term grazing strategies (Dowling et al. 2005). In Australia, the most widely adopted grazing practice is continuous grazing or set‐stock grazing, where a single flock of animals is restricted to one paddock for an extensive period (Mavromihalis et al. 2013; Sato et al. 2019). Under a set‐stock grazing regime there is higher potential for livestock to selectively overgraze preferred areas of the grassland, which over time can lead to a reduction in desirable plant species. Rotational grazing utilises high‐intensity short‐duration grazing in which stock rotate through multiple paddocks which typically spreads grazing pressure more uniformly across the landscape (Teague et al. 2013). Rotational grazing aims to maximise carrying capacity by increasing grazing pressure on less‐desirable areas (i.e., low palatability plants) and reducing pressure on preferred (i.e., high palatability) areas. Lawrence et al. (2019) found consistent long‐term rotational grazing significantly increased foliar cover of perennial herbaceous species, reduced introduced annual plant species and enhanced biophysical function related attributes. However, rotational grazing can decrease landscape heterogeneity and adversely affect soil properties without appropriate intensive management by the landowner (Teague et al. 2013). As such, further research is needed to better understand the net effects of rotational grazing on biodiversity across different landscape and management contexts.

In Australia, livestock grazing is generally considered to have degradative effects on ecosystem function and biodiversity (Eldridge et al. 2016). Livestock grazing can affect multiple trophic levels and thus drive biodiversity and biophysical patterns within a landscape. Changes in vegetation structure are largely associated with landscape management history and climatic conditions (Price et al. 2021), but in agricultural grasslands grazing is widely considered a management tool to prevent native species decline and exotic species infestation beneficial for both livestock and endemic fauna (Marriott et al. 2009; Souter and Milne 2009; Pakeman et al. 2019). Animals directly dependent on plants typically exhibit a stronger negative association with livestock presence, whereas detritivores are more positively associated with livestock (Filazzola et al. 2020). However, the cumulative effects on vegetation, soil, and lower trophic levels are expected to have a greater impact on some fauna than grazing alone (Dorrough et al. 2012; Rotem et al. 2016). For example, typically reptile abundance and richness decrease with increasing grazing intensity (Howland et al. 2014; Val et al. 2019). However, individual species have shown positive and negative responses to grazing pressure (Dorrough et al. 2012; Howland et al. 2014; Neilly, Nordberg, et al. 2018). This is further complicated by species that respond negatively in the short term but ultimately require grazing or alternative management for long‐term persistence (Scroggie et al. 2019).

**FIGURE 1:**
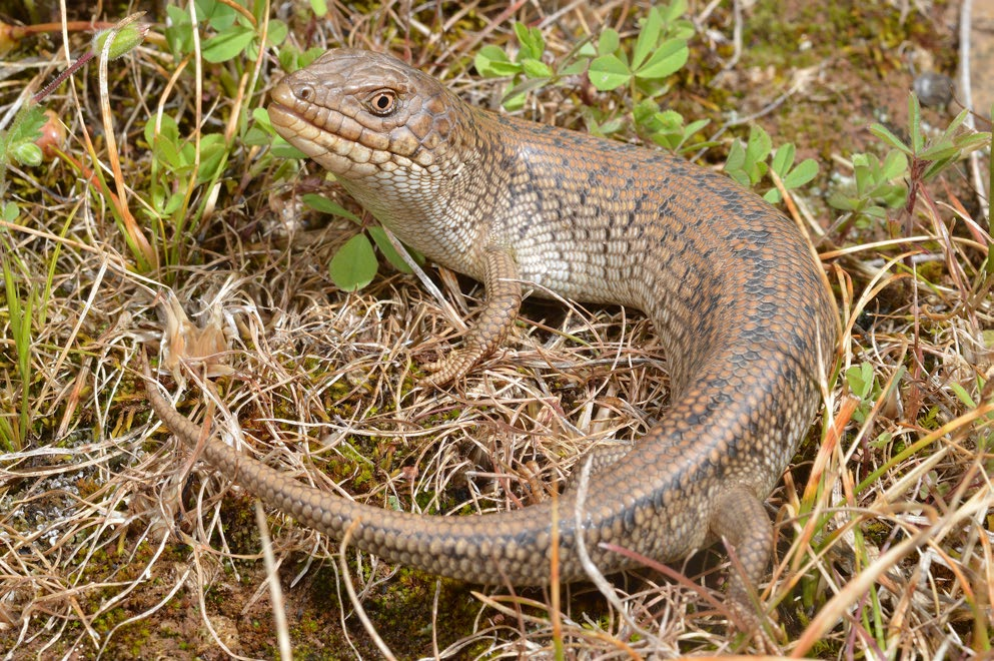
Image of pygmy bluetongue lizard. Photo provided by Mike Swan.

Here, we assessed the effects of productive grazing practices on the endangered pygmy bluetongue lizard (*Tiliqua adelaidensis*) and habitat quality. Pygmy bluetongues are known to occur only on private grazing properties predominantly grazed by sheep within a restricted range of the Mid North region of South Australia and are dependent on compatible grazing regimes for their survival (Gardner 2024). The lizards are closely associated with burrowing spiders and occupy vacant spider burrows (Milne and Bull 2000) (Figure 1). This study was part of a long‐term grazing project collaborating with landowners to erect new fence lines to facilitate effective livestock grazing management for pygmy bluetongue conservation. We compared the short‐term effects of changing grazing regime from set‐stock to rotationally grazed, and grazing exclusion, against an existing rotationally grazed area on the same property. Rotational grazing is being implemented increasingly throughout the pygmy bluetongue range. While some form of grazing to maintain vegetation structure is conducive for pygmy bluetongue habitat, a high livestock density has the potential to trample spider burrows that the lizards inhabit (Clayton and Bull 2016). We investigated a property with the most northerly known population of pygmy bluetongues and where lizards are negatively associated with high areas of bare ground cover on a microhabitat‐ (Michael et al. 2024a) and site‐scale (Michael et al. 2025). Further, this population is most at risk of climate change induced habitat alteration and local extinction of pygmy bluetongues (Delean et al. 2013; Michael et al. 2024b). We hypothesised rotational grazing would increase vegetation cover, reduce trampling of spider burrows, and have a positive effect on lizard abundance and body condition compared to set‐stock grazing.

## 2 Methods

This study was conducted near the township of Peterborough approximately 300 km north of Adelaide. Peterborough has a hot and dry climate and the mean annual rainfall in Yongala (approx. 10 km from the study site) from 1991 to 2020 was 353.5 mm (Bureau of Meteorology 2024). However, the region was in drought for the study period with a mean annual rainfall of 251.9 mm (Bureau of Meteorology 2024). The study site is a privately owned sheep grazing property as are most agricultural properties near Peterborough. The property predominantly uses two forms of grazing—rotational and set‐stock grazing. There is a system of nineteen rotational grazing paddocks that are approx. 172 ha each and sheep remain within a paddock for approximately 1 month before being moved. The set‐stock paddock is approximately 460 ha and sheep remain for approximately 10 months of the year. Pygmy bluetongues are found in both rotational and set‐stock grazed areas, but it is not clear if the lizards are able to move between the two grazed areas as they are divided by a road. On the property, the areas that the lizards inhabit have less vegetation cover and a sparser mixture of native tussocks, exotics and other ground cover species compared to other pygmy bluetongue‐occupied sheep properties (Michael et al. 2024b, 2025).

**FIGURE 2:**
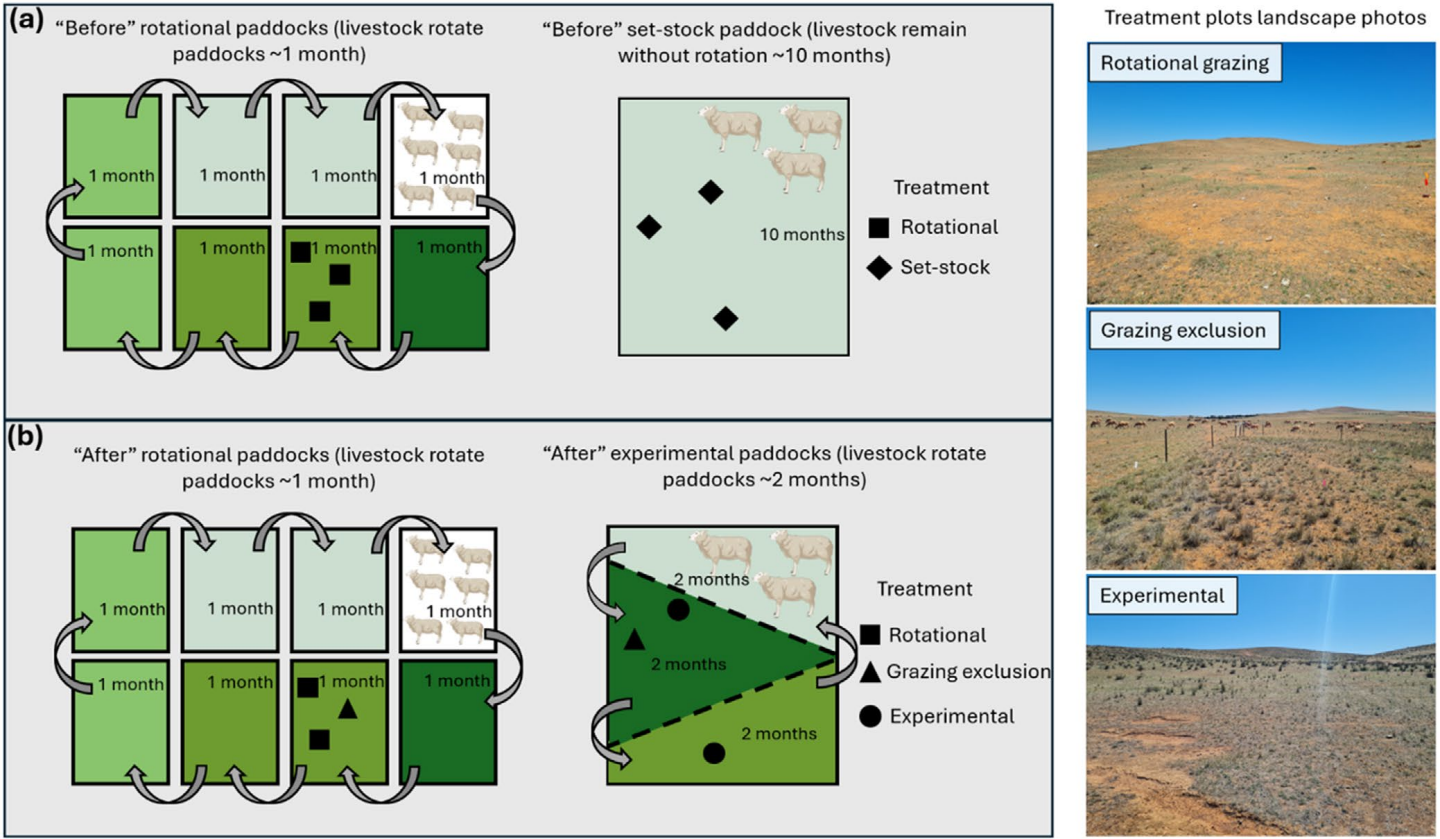
Graphic illustrating experimental design of treatment plots in the landscape in (a) ‘before’ (November 2022–April 2023); (b) ‘after’ (September 2023–November 2024) surveys. Rectangle outlines represent fenced paddocks; arrows indicate movement of livestock between paddocks; dashed lines indicate erected fence lines. Pasture recovery indicated by shading. Example ‘after’ (November 2024) photos of treatment plots. Figure is not to scale.

We used a ‘before’ and ‘after’ comparative study design to investigate the effects of grazing treatment on pygmy bluetongues, spider burrows and habitat quality. The study was conducted over 2 years using six 30 × 30 m plots. All plots were spaced a minimum of 100 m apart in areas inhabited by pygmy bluetongues. Three plots were established in an area that had been rotationally grazed for more than a decade, and three plots were established in the set‐stock area in ‘before’ surveys (Figure 2a). ‘Before’ surveys occurred in November 2022 and April 2023. New fence lines were erected in February 2023 to subdivide the set‐stock area into three rotational grazing paddocks, and in June 2023 two plots (one rotational and one set‐stock) had grazing exclusion fences erected. Therefore, in ‘after’ surveys there were two rotational grazing, two grazing exclusion and two experimental grazing plots (where grazing had changed from set‐stock to rotational) (Figure 2b). ‘After’ surveys occurred in September 2023, November 2023, February 2024, March 2024 and November 2024. During all survey periods, vegetation, spider burrow, lizard abundance and body condition surveys were conducted, except in September 2023 where only vegetation and lizard abundance surveys were undertaken and February 2024 where only body condition was assessed. Stock levels were not experimentally manipulated away from regularly occurring farming regimes; thus, stock movements were managed by the landowner.

### 2.1 Pygmy Bluetongue and Spider Burrow Surveys

Within each plot, we walked 30 one‐metre‐wide transects north–south and 30 one‐metre‐wide transects east–west, marking every arthropod and spider burrow found. Each burrow was then inspected with a Yateks M615FM optiscope. We measured the total depth of every burrow and recorded what fauna was occupying the burrow with the abundance of pygmy bluetongues recorded within all plots during each survey period. To assess body condition of lizards, we lured lizards from their burrow following Milne and Bull (2000), measured their snout‐vent length to the closest millimetre, and weighed to the nearest gram. Caught lizards were toe clipped to assign a unique identifying number.

### 2.2 Vegetation Surveys

We assessed landscape structure on an intermediate scale by using three 30 m transects per plot, spaced evenly apart, starting from the southern boundary of plots. Along each transect we conducted a line point intercept survey starting at 0 m where we recorded what the transect intercepted with (e.g., vegetation, bare ground, leaf litter, rock, moss, lichen) at every 3 m point. On a finer vegetation habitat scale we randomly placed a 1 × 1 m quadrat, identified every plant species to the lowest taxonomic group possible, usually species (Mid North Grasslands Working Group 2006), measured the height of each species, and estimated percent cover to the nearest 5% within each quadrat. For statistical analyses, we grouped identified plant species into the functional groupings of ground covers (e.g., *Trifolium* spp.), tussocks (e.g., *Aristida behriana*) and exotic plants (e.g., *Silybum* spp.).

### 2.3 Statistical Analysis

We subset the data into ‘before’ data prior to the erection of any new fence lines (i.e., rotational versus set‐stock) (November 2022 and April 2023) and ‘after’ data post erection of fence lines and grazing exclusion plots (i.e., rotational versus experimental versus grazing exclusion) (September 2023, November 2023, February 2024, March 2024 and November 2024) and conducted analyses in R statistical software (R Core Team 2024) and Primer/PERMANOVA+ (v.7.0.13). We chose to analyse the ‘before’ and ‘after’ data separately to account for changes in treatments, number of surveys and natural environmental variation, which required different models and variables for each phase.

We used generalised linear models (GLMs) to detect any relationship between pygmy bluetongue abundance and a suite of uncorrelated vegetation variables in ‘before’ and ‘after’ datasets. To determine which correlated variables to remove to obtain the suite of uncorrelated variables we firstly checked variables with > 30 observations for collinearity (Pearson's rank > 0.60) and then performed a Principal Component Analysis (PCA). We retained variables with the highest loadings on PC1 and PC2 that captured > 70% of the variance in the data. Due to the difference in treatments in ‘before’ and ‘after’ surveys, and our process to refine the number of covariates prior to modelling, the ‘before’ and ‘after’ models had a different set of covariates. We standardised the uncorrelated vegetation variables prior to analysis, included grazing treatment and its interaction with survey period, and used a Poisson distribution for both ‘before’ and ‘after’ models. We did not include Plot as a random factor as preliminary analyses showed minimal variation at the plot level that had only six replicates, and instead variation was better explained at the treatment and vegetation‐variable level (Oberpriller et al. 2022).

The body condition index was calculated as the residuals of the linear regression of log body mass on log snout‐vent length (log_e_ BM, log_e_ SVL; Shamiminoori et al. 2014). Only adult lizards (> 80 mm snout‐vent length) were used for body condition analyses. We did not attempt to differentiate between male and female lizards in our analyses as lizard sex is difficult to determine outside the breeding season, but we noted the sex of specific individuals during the breeding period. We used a linear mixed‐effect model for both ‘before’ and ‘after’ datasets. In both models, we included grazing treatment and its interaction with survey period, and lizard identifier as a random factor to account for repeated sampling of the same pygmy bluetongues over time. In ‘after’ surveys, the second body condition survey (February 2024) was used as the reference time point rather than the first (November 2023) survey. This decision was based on high variability observed in female body condition during the breeding season (early‐late summer). Pygmy bluetongues may have up to four live young, and during this sampling period some females were caught with babies within their maternal burrows, while others were still gravid. The number of lizards used for abundance and body condition analyses is available in Table S1.

To assess whether mean spider burrow depth was influenced by grazing treatment we used generalised linear mixed‐effect models (GLMM) with a gamma log‐link function. In both ‘before’ and ‘after’ models we included grazing treatment and its interaction with survey period and plot as a random factor.

To visualise vegetation structural differences between season and grazing treatment, we conducted a Canonical Analysis of Principal (CAP) coordinates modelled approach for ‘before’ and ‘after’ datasets. For ease of visual interpretation, we plotted variables with a correlation value of > 0.4. We also analysed the effect of grazing treatment in the ‘after’ dataset on vegetation cover with a GLM fitted with a Poisson distribution.

## 3 Results

### 3.1 Pygmy Bluetongue Abundance

The language used to describe statistical significance was derived from Muff et al. (2022). There were minimal effects of vegetation structure, grazing treatment or time on pygmy bluetongue abundance in ‘before’ data (Table 1). Vegetation cover and grazing exclusion both had a significant positive effect on lizard abundance in ‘after’ data. Each of the time points (November 2023, March 2024, November 2024) also showed moderate–strong significant effects. Interaction terms between the grazing exclusion and time points were also moderate–strongly significant (Table 2).

**TABLE 1:** Results of generalised linear model for pygmy bluetongue abundance in relation to vegetation structure in ‘before’ data.

Model	Coefficient	Estimate	SE	Wald chi‐squared statistic	*p*
‘Before’	Intercept	1.95	0.54	3.60	**< 0.001**
	Leaf litter	0.32	0.60	0.54	0.590
	Tussock height	0.17	0.23	0.72	0.471
	Ground cover height	0.16	0.31	0.54	0.590
	Exotic	−0.16	0.29	−0.55	0.584
	Exotic height	0.16	0.32	0.49	0.624
	Set‐stock grazing	−1.21	0.66	−1.84	0.066
	April 2023	−0.08	0.99	−0.08	0.939
	Set‐stock*April 2023	−0.22	0.79	−0.28	0.780

*Note:* Reference states for comparison were rotational grazing plots and November 2022. Bold indicates *p* < 0.05.

Abbreviation: SE, standard error.

**TABLE 2:** Results of generalised linear model for pygmy bluetongue abundance in relation to vegetation structure in ‘after’ data.

Model	Coefficient	Estimate	SE	Wald chi‐squared statistic	*p*
‘After’	Intercept	−0.82	0.80	−1.02	0.308
	Vegetation	0.88	0.34	2.59	**0.010**
	Bare ground	−0.23	0.34	−0.68	0.499
	Ground cover height	0.41	0.22	1.82	0.068
	Tussock height	0.39	0.31	1.25	0.211
	Exclusion	1.80	0.61	2.97	**0.003**
	Experimental	−0.19	0.88	−0.22	0.828
	November 2023	3.53	1.45	2.43	**0.015**
	March 2024	3.58	1.41	2.53	**0.011**
	November 2024	3.20	1.10	2.93	**0.003**
	Exclusion*Nov‐23	−3.00	1.24	−2.41	**0.016**
	Experimental*Nov‐23	−3.43	1.42	−2.42	**0.016**
	Exclusion*Mar‐24	−2.84	1.08	−2.63	**0.009**
	Experimental*Mar‐24	−3.48	1.55	−2.25	**0.025**
	Exclusion*Nov‐24	−3.15	1.06	−2.97	**0.003**
	Experimental*Nov‐24	−1.10	1.27	−0.87	0.386

*Note:* Reference states for comparison were rotational grazing plots and September 2023. Bold indicates *p* < 0.05.

Abbreviation: SE, standard error.

### 3.2 Body Condition

**FIGURE 3:**
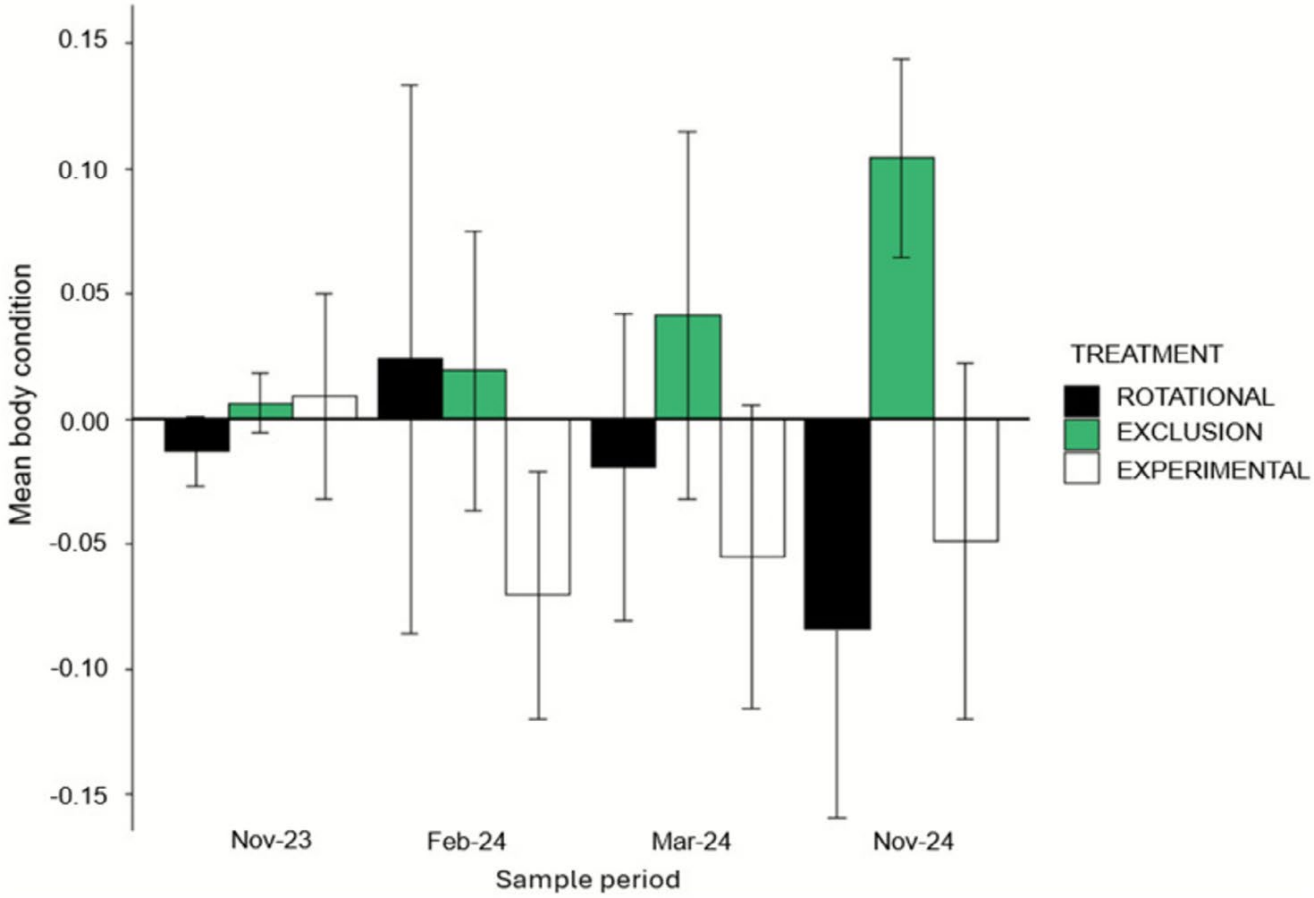
Mean (SE) body condition of adult pygmy bluetongues in ‘after’ data across the three treatments.

The linear mixed‐effects model showed there was no evidence for a combined effect of grazing treatment or time on body condition in ‘before’ data (Table S2). In comparison, we found a moderate positive effect only for the interaction between grazing exclusion and November 2024 in the ‘after’ body condition model (Figure 3). No other variables or interactions showed significant effects in this model (Table S3).

### 3.3 Spider Burrows

**FIGURE 4:**
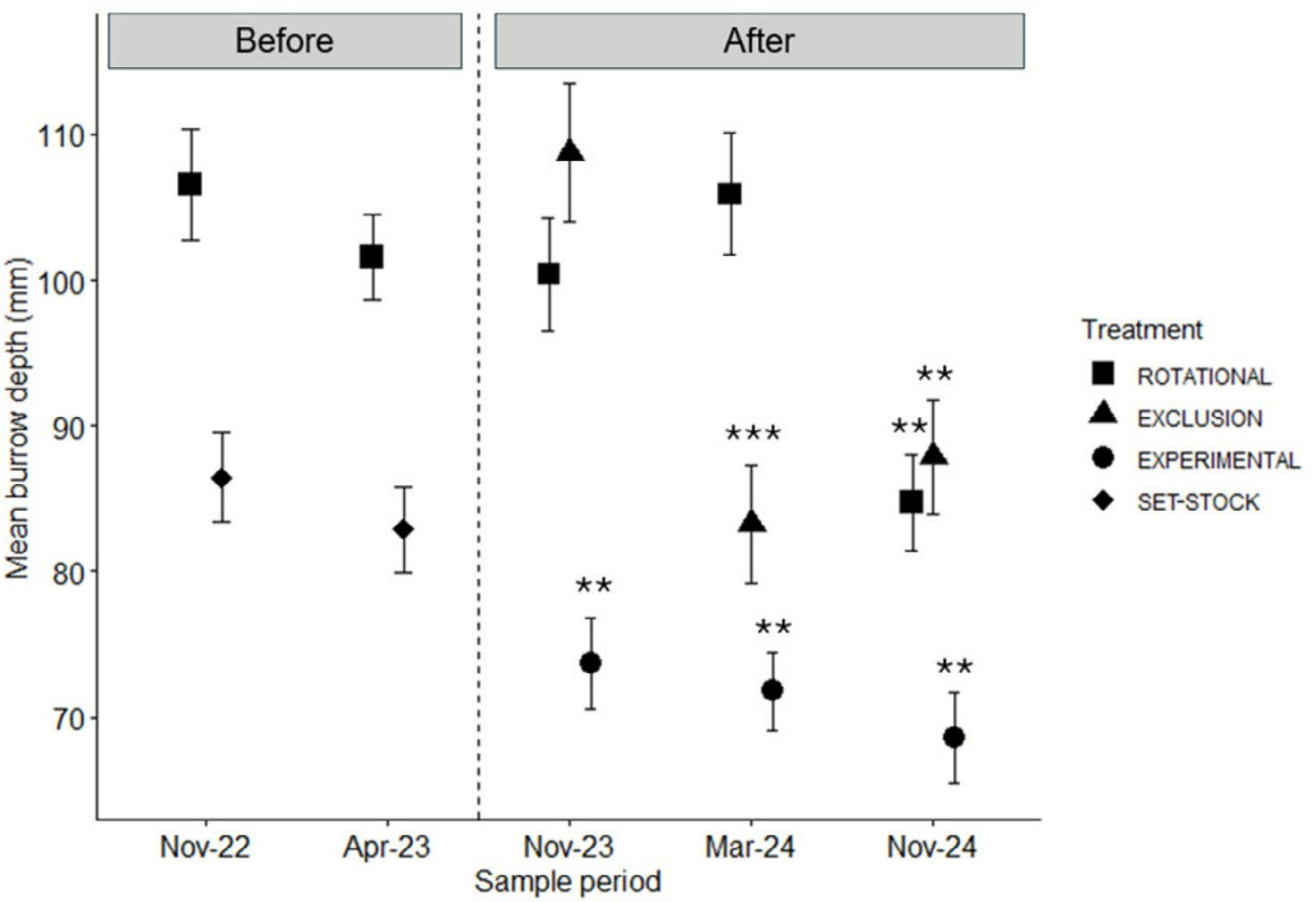
Mean (SE) burrow depth during ‘before’ (rotational and set‐stock) and ‘after’ surveys (exclusion, experimental and rotational). Asterisks denote significant differences in mean burrow depth (*p < 0.01; **p < 0.001; ***p < 0.0001).

We did not detect any significant effect of grazing treatment, time or their interaction; but there was a significant effect on the intercept, on mean burrow depth in ‘before’ surveys (Table S4; Figure 4). In ‘after’ burrow surveys, we found strong evidence burrows in the experimental grazing area were shallower on average, very strong evidence burrows in grazing exclusion areas in March 2024 were shallower, and strong evidence burrows in all grazing treatments surveyed in November 2024 were shallower (Table S5, Figure 4).

### 3.4 Vegetation

**FIGURE 5:**
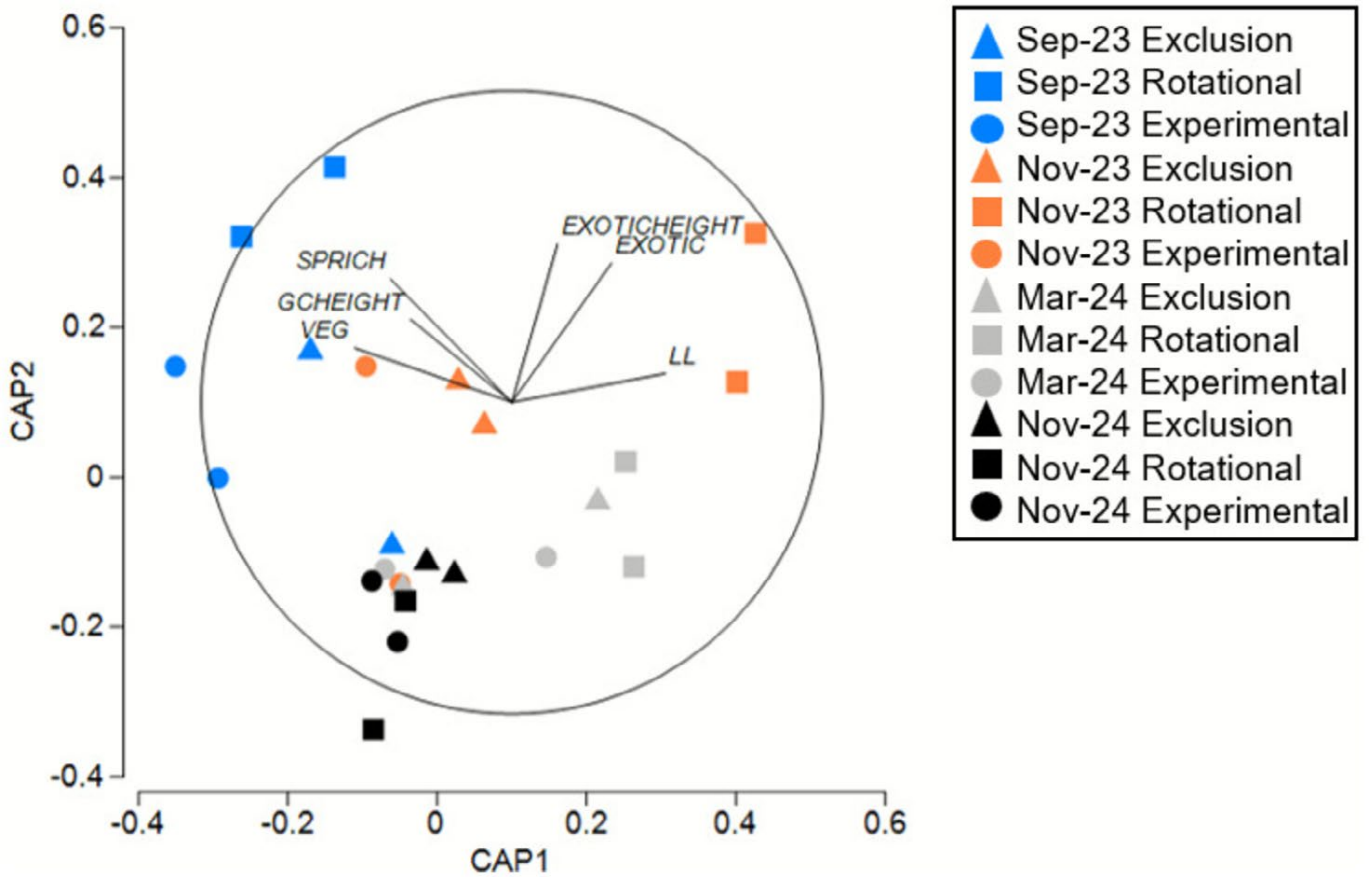
Canonical analysis of principal coordinates (CAP) plot showing canonical axes that best discriminate vegetation structure in ‘after’ data among grazing treatment and season (> 0.4 correlation). SPRICH: species richness, GCHEIGHT: average height of ground cover plants, VEG: line point intercept count of vegetation, EXOTICHEIGHT: average height of exotic plants, EXOTIC: average ground cover percentage of exotic plants, LL: line point intercept count of leaf litter.

We assessed vegetation structural differences among grazing treatments and seasons in ‘before’ and ‘after’ surveys. The ‘before’ CAP did not show distinct separation in vegetation structure between rotational and set‐stock grazed areas or seasons (tr(Q_m'HQ_m): 1.57, *p* = 0.266, delta 1^2^: 0.93, *p* = 0.116, 999 permutations) (Figure S1). In comparison, the ‘after’ CAP showed separation between the grazing treatments (rotational, experimental, exclusion) and seasons (tr(Q_m'HQ_m): 2.529, *p =* 0.01, delta 1^2^: 0.916, *p* = 0.049, 999 permutations) (Figure 5). While seasonal differences are more evident, as points from the same sample periods tend to cluster closer together, the grazing treatments showed more variability within each season. However, in more recent surveys (March and November 2024), all three grazing treatments were more closely clustered compared to earlier seasonal periods.

There was strong evidence for ‘after’ experimental and weak‐moderate evidence for grazing exclusion plots to have greater vegetation cover compared to rotational grazing plots (Table 3).

**TABLE 3:** Results of generalised linear model for ‘after’ vegetation cover in relation to grazing treatment.

Model	Coefficient	Estimate	SE	*p*
Vegetation cover (‘after’)	Intercept	1.891	0.137	**< 0.0001**
	Grazing exclusion	0.347	0.179	0.053
	Experimental	0.461	0.175	**0.009**

*Note:* Reference states for comparison were rotational grazing plots. Bold indicates *p* < 0.05.

Abbreviation: SE, standard error.

## 4 Discussion

The impacts of livestock grazing on native fauna may be positive or negative and depend upon grazing history, grazing intensity, productivity and climate (Neilly, O'Reagain, et al. 2018). Pygmy bluetongues have exhibited negative (Nielsen and Bull 2020), positive (Pettigrew and Bull 2012) and neutral (Michael et al. 2025) responses to livestock grazing. In this study, we used a ‘before’ and ‘after’ comparative design to investigate the impact of changing livestock management from a set‐stock grazed system to rotationally grazed and grazing exclusion. We detected positive lizard abundance and body condition responses in ‘after’ grazing exclusion plots and found strong evidence ‘after’ vegetation cover increased in the experimental plots and weak‐moderate evidence there was also an increase in grazing exclusion plots. These results represent possible co‐benefits to conservation and agriculture where increased vegetation cover, because of changed grazing regime, will likely lead to long‐term benefits for both pygmy bluetongues and sheep by improving lizard habitat quality and forage availability for livestock (Teague et al. 2013; Mulhall et al. 2022; Porensky et al. 2020).

### 4.1 Grazing Impacts on Pygmy Bluetongues

Pygmy bluetongues exhibit distinct habitat associations at multiple spatial scales depending on the quality of habitat available. At high quality landscapes where vegetation cover is greater, the lizards typically do not show distinct associations at a microhabitat‐ or site‐scale (Michael et al. 2024a, 2025). However, at lower quality sites where vegetation cover is sparse (i.e., Peterborough), pygmy bluetongues prefer greater vegetation cover at both the microhabitat‐ (Michael et al. 2024a) and site‐scale (Michael et al. 2025). Our results are congruent with previous findings, and here, we show that pygmy bluetongues are positively associated with greater vegetation cover on a landscape‐scale at the same lower quality site (i.e., Peterborough). Lizard abundance on average across all sample periods was positively associated with the grazing exclusion plots but not the experimental plots despite vegetation cover increasing in both treatments. This result may have been driven by the presence of neonates and deeper burrows within grazing exclusion plots. Adult pygmy bluetongues are typically associated with one burrow and remain within their burrow throughout the year except during the spring mating season (Bull et al. 2015), whereas neonates will remain in the maternal burrow for a short duration before dispersing (Milne et al. 2002). During this study, adult lizards typically remained within the same burrow across all survey periods and neonates were detected in maternal burrows across all grazing treatments. However, only neonates in grazing exclusion plots were recorded across multiple consecutive surveys (i.e., established independent burrow). Neonates remaining in areas of greater vegetation cover may indicate that in addition to burrow availability, greater vegetation cover influences reduced dispersal distance of neonates.

The sample period and grazing treatment interacted negatively with pygmy bluetongue abundance in both experimental and exclusion treatments relative to the rotational grazing area. These results suggest the short‐term impacts of these treatments did not outweigh the long‐term effects of established rotational grazing on pygmy bluetongues that have been in effect for more than a decade. A significant time‐lag in pygmy bluetongue response would be expected following changes in grazing regime for vegetation structure changes to fully accumulate (O'Sullivan et al. 2023). Continued agricultural management that promotes vegetation cover, such as rotational grazing, is likely to promote long‐term benefits for the pygmy bluetongue, as vegetation cover is an indicator of habitat suitability for other fossorial species (Wong et al. 2021).

### 4.2 Body Condition

Pygmy bluetongue body condition has been previously shown to be positively correlated with increased vegetation cover (Fenner and Bull 2007) and the lizards are typically in their best body condition in early summer (October–December) (Shamiminoori et al. 2014). We found lizard body condition was greater in the grazing exclusion final November 2024 survey compared to rotational and experimental plots. Our results showed a positive trend for body condition to increase over time in grazing exclusion plots above the expectations of peak body condition associated with early summer. Pygmy bluetongues are ambush predators of terrestrial arthropods that pass by their burrow entrance (Milne and Bull 2000; Souter et al. 2007), and increased vegetation cover is positively associated with terrestrial arthropods (Bromham et al. 1999; Houston et al. 2023). Therefore, the increased vegetation cover within grazing exclusion plots was likely positively associated with an increase in prey availability for lizards. Vegetation also increased in experimental plots although body condition was not found to increase. Male pygmy bluetongues have a higher body condition than females (Shamiminoori et al. 2014) and therefore our results may have been skewed by unequal proportions of male lizards as experimental plots lacked males.

### 4.3 Spider Burrows

Burrows in ‘after’ surveys were shallower on average in the experimental plots possibly because the new fence lines restricted sheep movement within experimental plot areas for longer and exacerbated trampling effects. Burrows in the grazing exclusion plots were shallower in March 2024. We expected burrow depth to increase where grazing was removed; however, changes at the microhabitat‐scale facilitated by grazing exclusion may have affected the arthropod community. An increase in vegetation cover is positively associated with terrestrial arthropods (Bromham et al. 1999; Houston et al. 2023), which may have led to an increase in arthropod abundance and their initial burrow digging. Burrow digging is a time intensive process by arthropods to achieve deep burrows (Suter et al. 2011), therefore, the overall decrease in depth may reflect an increase in new, shallow burrows. We also found burrows in all grazing treatments to be shallower in the final November 2024 survey. This finding may be a result of a flood event during the same month. Burrows that are unoccupied or surrounded by less vegetation are more easily destroyed by heavy rainfall (Ebrahimi et al. 2012). Ultimately, we found no direct positive effect of grazing treatment on spider burrow depth, consistent with Clayton and Bull (2016). Identifying impacts on burrows may take time due to livestock presence, climate interactions and spider reproductive cycles.

### 4.4 Vegetation

Grazing removal had a weak‐moderate positive effect on grazing exclusion plots. Effects of changes in grazing regime are context‐dependent and vary across gradients of soil fertility and rainfall (Schultz et al. 2011). A previous grazing removal study in the Mid North region of South Australia found a similar result where grazing removal increased basal vegetation cover (Souter and Milne 2009). Our increased vegetation cover result in the grazing exclusion plots concurs with those results, but we did not investigate species richness changes as pygmy bluetongues are not associated with a specific species of plant, rather the morphological vegetation structure (Souter et al. 2007). Further, species composition as an indicator of change is less sensitive than bare ground or basal area (Teague et al. 2004). Notably, we previously assessed changes in bare ground cover within 10 months of grazing exclusion fence erection and found no significant difference in bare ground cover before or after grazing exclusion (Michael et al. 2025). There appears to be a time‐lag for vegetation structural differences to accumulate, given we identified significantly more vegetation cover, and therefore a decrease in bare ground cover, in the same plots but 18 months post grazing exclusion (O'Sullivan et al. 2023). To fully understand the cumulative vegetation responses to grazing, a longer time period, such as at the decadal scale, is required (James et al. 2001; Michael et al. 2022).

Vegetation cover increased when grazing was changed from set‐stock to rotational grazing. Rainfall, soil carbon levels and livestock dietary preferences collectively influence pasture composition and quality, thus it is difficult to discern the impact of grazing regime changes on vegetation structure alone (Paine et al. 1999; Rotem et al. 2016). Our study coincided with below average rainfall, which likely had an interactive effect with grazing regime on vegetation structure. However, it is possible the change in grazing regime decreased the rate of deterioration, which led to an increase in vegetation cover (Teague et al. 2004). The increase in vegetation cover likely increased forage availability for livestock, but further investigation is warranted to understand whether forage quality or composition were affected.

Our ‘after’ CAP analysis indicated that vegetation structure among the three grazing treatments became more uniform over time, supported by the increase in vegetation cover in experimental plots. There is evidence that various forms of rotational grazing increase biophysical factors leading to improved forage quality and increased abundance of higher quality forage species compared to continuous or set‐stock grazing (Paine et al. 1999; Lawrence et al. 2019), which may have resulted in convergence of vegetation responses among grazing treatments in our most recent surveys. However, another comparative study found grass productivity was similar among rotational and set‐stock grazing regimes, but livestock weight gain was greater under set‐stock grazing management (Augustine et al. 2020). While we did not assess the profit margin for the landowner in this study, owing to the short timeframe and economic factors during the study period, the change in grazing regime has been well received by them. The landowner stated, ‘I always try to achieve one positive thing each year on the farm and this year was those fence lines. It's easier to muster the sheep, closer to water and better grazing pressure’ (Pers. Comm. Mark Ludgate, 15/04/2024). This perspective demonstrates how conservation and agriculture can complement one another, with practices such as rotational grazing improving both livestock management and habitat quality. In our study, this collaboration not only supported better outcomes for the landowner but also improved habitat quality for the pygmy bluetongue. Building such partnerships is a crucial step toward ensuring the long‐term persistence of threatened species, as they foster trust, encourage future research access and promote the spread of conservation practices across the wider community. Our study implemented a landowner‐dictated stocking rate rather than a manipulation to provide a ‘typical’ use case, further facilitating the cooperation of landowners.

### 4.5 Implications

Although we found pygmy bluetongue abundance and body condition were positively associated with grazing exclusion, we caution extrapolating this result to a landscape‐scale. The long‐term effects of grazing exclusion are yet to be explored on this species, but pygmy bluetongues have coexisted with some form of grazing likely for millennia and with livestock grazing since the inception of livestock farming in the Mid North of South Australia (Gardner 2024). Livestock contribute to landscape heterogeneity and their long‐term exclusion may negatively impact lizards' ability to effectively thermoregulate and catch prey. Short‐term grazing exclusion could be considered on lower quality sites to provide a temporary buffer from trampling by livestock while increasing habitat quality and prey availability during drought periods with the effects likely to be greater during drier periods such as experienced during the study. On other properties, the implementation of rotational grazing could also have substantial benefits for pygmy bluetongues and livestock grazing management (Gardner 2024).

The net effects of grazing on vulnerable species, such as the pygmy bluetongue, are difficult to establish without using manipulative grazing experiments that experimentally control stocking rates. However, ensuring that agricultural activities were maintained at a productive level by the landowner allowed for more accurate insights into the effects of grazing for the species on a broader scale and provided rare and valuable ‘before’ and ‘after’ ecological data that fostered a long‐term collaborative partnership with a landowner who will continue to allow access to the property for further research. The study design was limited by the employment of a mensurative approach to stocking rates rather than manipulative. Stocking rates and grazing rotations are influenced by local climate and economic conditions by the landowner (Dubois and Carson 2020), and alterations to livestock management in future years guided by local conditions could affect vegetation cover and pygmy bluetongue associations. Due to the logistical constraints of reduced pygmy bluetongue population area aligned with agricultural land management, the study design has limited statistical power to detect small‐moderate effects due to the number of low replicates. Despite the low number of replicates, the observed vegetation and pygmy bluetongue responses were strong and followed similar observed patterns across other spatial scales at the same property (Michael et al. 2024a, 2025). Increasing replicates would increase statistical power to detect small‐moderate effects and provide greater ecological insights into vegetation and pygmy bluetongue responses to landowner‐led livestock management. Continuing to investigate mensurative approaches to landowner‐led livestock management is critical to ensure any extrapolations of ecological interactions that occur between threatened species and livestock on private properties support broader regional extrapolation of results. Similar studies should consider increasing replication and temporal scale to better capture small‐moderate effects and different local climate conditions.

Reptiles respond to their environment at multiple spatial scales (Fischer et al. 2004; Michael et al. 2010) and microclimates (Doucette et al. 2023) and therefore managing livestock grazed landscapes to ensure appropriate refugia for all inhabiting species is a key challenge. While we did not assess multiple spatial scales in this study, we build upon our previous work that investigated the microhabitat‐ (Michael et al. 2024a) and site‐ (Michael et al. 2024b, 2025) scales at the same property to now inform the landscape‐scale. Broadly, pygmy bluetongues have consistently been found to be positively associated with increased vegetation cover at all three spatial scales (i.e., microhabitat, site, landscape) at the northernmost known population. However, when compared to other climatically variable properties that consist of greater vegetation cover than this property, pygmy bluetongues do not show a distinct association with vegetation cover (Michael et al. 2025). Our results are supportive of other studies—that the pygmy bluetongue responds to its environment at multiple spatial scales and may be influenced by climatic variations.

## 5 Conclusions

The perpetual conservation of species that inhabit private grazing land requires multidisciplinary research in conjunction with landowners adopting grazing strategies that improve land condition and by association improve habitat quality for biodiversity. Our results support that there was no trade‐off between pygmy bluetongue conservation and agricultural productivity (Neilly, O'Reagain, et al. 2018; Michael et al. 2025). Rather, increased vegetation cover because of implementing rotational grazing was conducive for both lizards and livestock management, demonstrating potential economic advantages for landowners. Successful collaborative initiatives that provide mutual benefits to both landowners and conservationists by protecting financial and ecological outcomes, such as this study, are integral to gain support from more landowners to allow similar research (Norris 2008; Neilly et al. 2016; Norton et al. 2020; Gardner 2024). This support will be vital for the pygmy bluetongue as translocation southerly is considered inevitable and, thus far, only privately owned sites have been identified as suitable for trial translocation (Michael et al. 2024b). Like many endangered species inhabiting private land, integrating agricultural practices with conservation initiatives offers a chance to not only maintain agricultural productivity and biodiversity, but potentially improve them too.

## Author Contributions

**Kimberley H. Michael:** conceptualization (equal), data curation (lead), formal analysis (lead), funding acquisition (equal), investigation (lead), methodology (lead), writing – original draft (lead), writing – review and editing (lead). **Patrick Michael:** data curation (supporting), investigation (supporting), supervision (supporting), writing – review and editing (equal). **Ryan Baring:** formal analysis (supporting), supervision (supporting), writing – review and editing (equal). **Michael G. Gardner:** conceptualization (equal), funding acquisition (equal), supervision (lead), writing – review and editing (equal).

## Funding

This study was supported by the Biology Society of South Australia, Royal Society of South Australia, Australian Government Research Training Program Scholarship to K.H.M., the Northern and Yorke Landscape Board, and an Australian Research Council Linkage Grant (LP190100071) to M.G.G.

## Ethics Statement

This research was conducted under the Flinders Animal Welfare Committee Permit (AEC BIOL5017) and South Australian Department for Environment and Water Permit (G25011).

## Conflicts of Interest

The authors declare no conflicts of interest.

## Supporting information


**Appendix S1:** ece372716‐sup‐0001‐AppendixS1.docx.

## Data Availability

Data is archived in the Flinders University Repository of Open Access Data Sets and available at: https://doi.org/10.25451/flinders.30244060.
