# Supplementary material for: Rotational Grazing Supports Coexistence of the Endangered Pygmy Bluetongue and Livestock on a Productive Agricultural Property
Source: Ecol Evol. 2025 Dec 16;15(12):e72716. doi: 10.1002/ece3.72716 (PMC12706526; doi:10.1002/ece3.72716)
Supplement: Supplementary file 1 — Appendix S1: ece372716‐sup‐0001‐AppendixS1.docx. [file ECE3-15-e72716-s001.docx]

**Supplementary Material for**

Rotational grazing supports coexistence of the endangered pygmy bluetongue and livestock on a productive agricultural property

Table S1. Number of pygmy bluetongue lizards used for abundance and body condition analyses.

| Sample period | Treatment | Pygmy bluetongue abundance | Number of adult lizards caught for body condition analysis |
| --- | --- | --- | --- |
| November 2022 | Rotational | 20 | 15 |
|  | Set-stock | 6 | 6 |
| April 2023 | Rotational | 21 | 4 |
|  | Set-stock | 6 | 3 |
| September 2023 | Rotational | 8 | N/A |
|  | Exclusion | 11 | N/A |
|  | Experimental | 4 | N/A |
| November 2023 | Rotational | 5 | 5 |
|  | Exclusion | 11 | 6 |
|  | Experimental | 2 | 3 |
| February 2024 | Rotational | N/A | 4 |
|  | Experimental | N/A | 6 |
|  | Exclusion | N/A | 3 |
| March 2024 | Rotational | 7 | 5 |
|  | Experimental | 12 | 5 |
|  | Exclusion | 1 | 2 |
| November 2024 | Rotational | 8 | 7 |
|  | Exclusion | 15 | 8 |
|  | Experimental | 2 | 5 |

Table S2. Results of linear mixed effect model for pygmy body condition in “before” data. Reference states for comparison were November 2022 and rotational grazing.
SE: standard error

| Model | Coefficient | Estimate | SE | *P* |
| --- | --- | --- | --- | --- |
| “Before” | Intercept | -0.02 | 0.03 | 0.622 |
|  | Set-stock | 0.08 | 0.06 | 0.205 |
|  | April 2023 | 0.02 | 0.05 | 0.680 |
|  | Set-stock * April 2023 | -0.07 | 0.08 | 0.427 |

Table S3. Results of linear mixed effect model for pygmy body condition in “after” data. Reference states for comparison were February 2024 and rotational grazing.
SE: standard error, bold indicates *P* <0.05.

| Model | Coefficient | Estimate | SE | *P* |
| --- | --- | --- | --- | --- |
| “After” | Intercept | 0.02 | 0.06 | 0.773 |
|  | Exclusion | 0.01 | 0.08 | 0.912 |
|  | Experimental | -0.07 | 0.10 | 0.481 |
|  | November 2023 | -0.07 | 0.07 | 0.319 |
|  | March 2024 | -0.06 | 0.07 | 0.415 |
|  | November 2024 | -0.1 | 0.07 | 0.137 |
|  | Exclusion * November 2023 | 0.08 | 0.10 | 0.429 |
|  | Experimental * November 2023 | 0.15 | 0.11 | 0.181 |
|  | Exclusion * March 2024 | 0.09 | 0.09 | 0.358 |
|  | Experimental * March 2024 | 0.06 | 0.12 | 0.629 |
|  | **Exclusion * November 2024** | 0.19 | 0.09 | **0.041** |
|  | Experimental * November 2024 | 0.10 | 0.11 | 0.376 |

Table S4. Results of generalised linear mixed-effect model for mean spider burrow depth in “before” data. Reference states for comparisons were November 2022 and rotational grazing.
SE: standard error, bold indicates *P* <0.05.

| Model | Coefficient | Estimate | SE | *P* |
| --- | --- | --- | --- | --- |
| “Before” | **Intercept** | 4.67 | 0.09 | **<0.001** |
|  | Set-stock | -0.02 | 0.12 | 0.077 |
|  | April 2023 | -0.03 | 0.04 | 0.361 |
|  | Set-stock * April 2023 | -0.01 | 0.06 | 0.887 |

Table S5. Results of generalised linear mixed-effect model for mean spider burrow depth in “after” data. Reference states for comparisons were November 2023 and rotational grazing.
SE: standard error, bold indicates *P* <0.05.

| Model | Coefficient | Estimate | SE | *P* |
| --- | --- | --- | --- | --- |
| “After” | **Intercept** | 4.58 | 0.07 | **<0.001** |
|  | Exclusion | 0.10 | 0.09 | 0.278 |
|  | Experimental | -0.30 | 0.10 | **0.002** |
|  | March 2024 | 0.03 | 0.05 | 0.449 |
|  | **November 2024** | -0.14 | 0.05 | **0.004** |
|  | **Exclusion * March 2024** | -0.30 | 0.07 | **<0.001** |
|  | Experimental * March 2024 | -0.05 | 0.07 | 0.523 |
|  | Exclusion* November 2024 | -0.08 | 0.07 | 0.227 |
|  | Experimental * November 2024 | 0.08 | 0.08 | 0.315 |


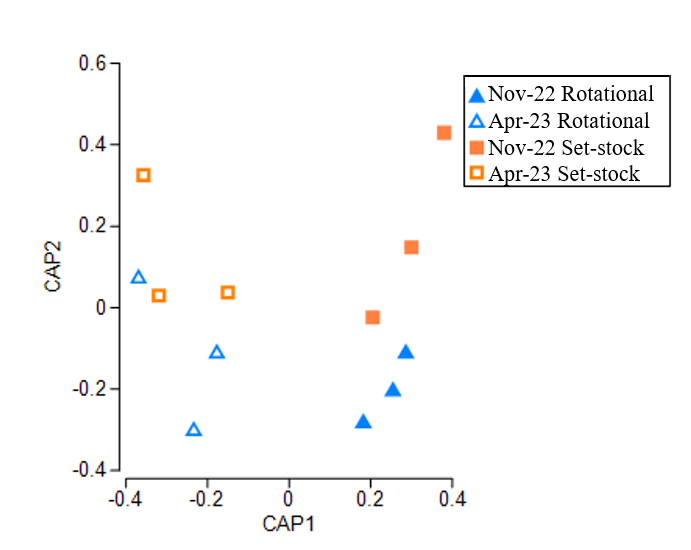


Fig S1. Canonical analysis of principal coordinates (CAP) plot showing canonical axes that best discriminate vegetation structure in “before” data among grazing treatment and season.
